# E-cadherin Expression in Premalignant Lesions, Premalignant Conditions, Oral Squamous Cell Carcinoma, and Normal Mucosa: An Immunohistochemical Study

**DOI:** 10.7759/cureus.44266

**Published:** 2023-08-28

**Authors:** Gnanambigai Kalaimani, Uma Devi K Rao, Elizabeth Joshua, Kannan Ranganathan

**Affiliations:** 1 Oral Pathology, Priyadarshini Dental College and Hospital, Thiruvallur, IND; 2 Oral Pathology and Microbiology, Ragas Dental College and Hospital, Chennai, IND; 3 Oral and Maxillofacial Pathology, Ragas Dental College and Hospital, Chennai, IND

**Keywords:** epithelial-mesenchymal transition, oral epithelial dysplasia, oral squamous cell carcinoma, oral potentially malignant disorders, e-cadherin

## Abstract

Background

Oral squamous cell carcinoma (OSCC) is a multi-step process. Epithelial-mesenchymal transition (EMT) is an important step in the progression of OSCC. One of the components that influence EMT is E-cadherin. The aim of this study was to determine the expression of E-cadherin in oral submucous fibrosis (OSMF), various grades of epithelial dysplasia, OSCC, and to compare it with the expression in the normal mucosa.

Material and methods

E-cadherin immunohistochemical detection was done using a monoclonal antibody of clone EP-6^TM^ and the PolyExcel HRP/DAB chromogen detection system. A total of 100 samples, were divided into four groups, which included epithelial dysplasia (group 2) (30 cases), oral submucous fibrosis (group 3) (OSMF-30 cases), and oral squamous cell carcinoma (group 4) (OSCC-30 cases), which was compared with normal mucosa (group 1) (10 cases). The positive control used for E-cadherin was ductal breast carcinoma.

Results

All the cases of normal mucosa, epithelial dysplasia, and OSMF showed positivity for E-cadherin expression. In OSCC, 97% of cases expressed E-cadherin except one case. Out of 30 cases of epithelial dysplasia, 53% of mild epithelial dysplasia had a moderate intensity of expression and 75% had a mild intensity of E-cadherin expression. In moderately differentiated OSCC, 82% of cases showed mild intensity. Tissue localization of the E-cadherin stain in the basal layer decreased from normal mucosa to grades of epithelial dysplasia and OSCC. The pattern of E-cadherin staining in all the cases of group I, group II, and group III was membranous. In 97% of OSCC cases, both membranous and cytoplasmic staining were seen.

Conclusion

E-cadherin expression was reduced in increasing grades of epithelial dysplasia, OSCC, and OSMF compared to that of normal mucosa. E-cadherin expression is reduced as the lesions progress to malignancy. Hence, E-cadherin can be considered a surrogate marker of malignancy.

## Introduction

Head and neck cancer (HNC) is one of the 10 most frequent cancers worldwide, with about 500,000 new cases being diagnosed annually. Oral squamous cell carcinoma (OSCC) shares part of HNC and has been reported to be increasing in betel quid chewing areas in recent years. Since most of them are not properly diagnosed in the beginning, oral cancers have the least five-year survival rates when compared to other cancers^.^[1].

Oral squamous cell carcinoma (OSCC) development is a multi-step process that necessitates the accumulation of numerous genetic abnormalities. Environmental factors, such as cigarettes, alcohol, chronic inflammation, and viral infection, as well as a patient's genetic predisposition, all play a role [2]. An epithelial-mesenchymal transition (EMT) is a biological process that enables a polarized epithelial cell to undergo numerous biochemical changes that enable it to assume a mesenchymal cell phenotype, which includes enhanced migratory capacity, invasiveness, elevated resistance to apoptosis, and greatly increased production of extra-cellular matrix components. Normally, the basal surface of the polarised epithelial cell interacts with the basement membrane to undergo these modifications. Epithelial cell-to-cell connections support cellular polarity and maintain tissue integrity [3].^ ^The junctional complex is composed of tight junctions, adherens junctions, and desmosomes. The adherens junction plays a pivotal role in regulating the activity of the entire junctional complex, and the major adhesion molecules in the adherens junctions are the cadherins [4]. From benign lesions to aggressive malignancy, loss of E-cadherin-mediated adhesion is a common feature [5]. OSCC often develops on the tongue, lips, and floor of the mouth [6]. It is believed that local invasion is a crucial initial stage in the spread of cancer, and it is thought that epithelial plasticity and EMT play a role in advancing the tumor [7].

 The purpose of the study was to investigate the expression of E-Cadherin in dysplasia, submucous fibrosis, and OSCC and compare it with normal mucosa to elucidate its role as a potential marker in determining the biological behavior of the disease.

## Materials and methods

Before the commencement of this study, an Institutional ethical committee approval was done. A total of 100 formalin-fixed and paraffin-embedded tissues were taken from the archives of the Oral Pathology department at Ragas Dental College and Hospital. Among these 30 cases were epithelial dysplasia (Group 2), 30 cases were OSCC (Group 4), 30 cases were OSMF (Group 33), and they were included in the study. Ten cases of normal mucosa (Group 1) were taken as controls. Tissue sections of ductal breast carcinoma were used as a positive control for E-cadherin positivity.

Immunohistochemistry

The antibody and reagents used for the IHC technique were obtained from VKan Life Care Private Limited, Chennai, and consisted of primary antibody, monoclonal rabbit anti-E-cadherin antibody (6 ml) clone EP-6; PolyExcel - HRP micro polymer anti-rabbit secondary antibody (6 ml) and substrate diaminobenzidine tetrahydrochloride (DAB) (1 ml). The sections of 5-micron thickness were deparaffinized in xylene and rehydrated by immersion in a graded series of alcohol. Slides were then antigen retrieved by treating with TRIS EDTA buffer of pH 9 at 15 lbs pressure for 15 minutes. Then, the slides were incubated with 3% hydrogen peroxide for five minutes to quench endogenous peroxidase activity and then the slides were dipped in TRIS buffer for 5 minutes. The pre-diluted primary antibody, monoclonal rabbit anti-E-Cadherin antibody (VKan Life Care Private Limited) was added and incubated for 30 minutes. The slides were then washed with TRIS buffer for 2-3 minutes and then the target binder reagent was added (15 min). Then, the slides were washed with TRIS buffer for 2-3 minutes and a drop of poly horseradish peroxidase was added and incubated for 15 minutes. For visualization, a drop of DAB was added for 10 min. Excess chromogen was removed with TRIS buffer and then counter-stained with hematoxylin. Then, the slides were dipped in increasing grades of alcohol and xylene. The slides were mounted with DPX and viewed under a light microscope. Ductal breast carcinoma was used as a positive control to ensure accurate and reproducible staining.

Immunohistochemical analysis

E-cadherin was assessed for epithelial and membranous localization. Then, each slide was graded as (-) nil or absence of stain, (+) mild, (++) moderate, and (+++) intensively stained based on the intensity of staining as observed by two blinded observers independently. The percentage of positive cells was also counted in each case, and it was categorized as 0 negative; 1+ = <10% of cells positive; 2+ =10% to 50%, and 3+ = >50%. Connective tissue was also examined in all the lesions.

The chi-square test was done to compare the expression of E-cadherin intensity and E-cadherin localization between the groups. P value <0.05 was considered statistically significant. Kappa analysis was done to compare the intensity of E-cadherin staining as observed between two observers.

## Results

The age of the patients was divided into three categories between the four groups: 20-40 years, 41-60 years, and those above 61 years of age. Group I consisted of nine (90%) cases in the age group 20-40 years and one (10%) case in the age group 41-60. Group II consisted of 12 (40%) cases in 20-40 years, 15 (50%) cases in 41-60 years, and three (10%) cases above 61 years. Group III consisted of 20 (67%) cases in 20-40 years, nine (30%) cases in 41-60 years, and one (3%) case above 61 years. Group IV consisted of eight (27%) cases in 20-40 years, 12 (40%) cases in 41-60 years, and 10 (33%) cases above 61 years of age (p=0.000). In group I, nine (90%) were males and there was one (10%) female. In group II, 22 (73%) were males and eight (27%) were females. In group III, 29 (97%) were males and one (3%) were females. In group IV, 21 (70%) were males and nine (30%) were females (p=0.03) (Table 1).

Based on the prevalence of habits in the study groups, they were categorized into seven groups. They were those without any habits, those with a habit of chewing tobacco and consuming alcoholic beverages, those chewing tobacco and smoking, those chewing tobacco alone, those smoking alone, those consuming alcoholic beverages and smoking, those chewing tobacco, and those consuming alcoholic beverages. In group I (the control group) none of them had any habits. In group II, one (3.33%) had the habit of chewing tobacco and consuming alcoholic beverages, three (10%) had the habit of chewing tobacco and smoking, two (7%) had the habit of chewing tobacco alone, seven (23%) had the habit of smoking alone, and 17 (57%) had the habit of smoking, chewing tobacco, and consuming alcoholic beverages. In Group III, four (13.33%) had the habit of chewing tobacco and smoking, 22 (74%) had the habit of chewing tobacco alone, and four (13.3%) had the habit of chewing tobacco, smoking, and consuming alcoholic beverages. In group IV, four (13%) had no habits, one (3%) had the habit of alcohol and chewing, three (10%) had the habit of chewing and smoking, two (7%) had the habit of chewing alone, six (20%) had the habit of smoking alone, three (10%) had the habit of consuming alcoholic beverages, and the habit of chewing, alcohol, and smoking seen in 11 (37%) (p=0.000) (Table 1).

In group I, 10 (100%) incisional biopsies were from the retro-molar region. In group II, 25 (83%) cases were from the buccal mucosa, four (14%) from the lateral border of the tongue, and one (3%) was from the labial mucosa. In group III, all the cases were from buccal mucosa. In group IV, 12 (40%) cases were from buccal mucosa, seven (23%) from alveolar mucosa, nine (30%) were from the lateral border of the tongue, and two (7%) from the labial mucosa (p=0.000) (Table 1).

The following parameters were used to evaluate E-cadherin staining in all four groups. Those were staining intensity, staining pattern, percentage of cells stained, and tissue localization of the stain. All the cases of normal mucosa showed membranous E-cadherin expression in the surface epithelia but not in the connective tissue. The immunostaining of E-cadherin was strong and homogenous in basal/suprabasal layers of the epithelium.

The grades of epithelial dysplasia were divided into three subgroups, namely, mild, moderate, and severe dysplasia. The E-cadherin expression in 19 cases of mild epithelial dysplasia was similar to that of normal epithelium. In moderate epithelial dysplasia, E-cadherin expression was present in the supra-basal layer but the expression was reduced in the basal cell layer. One case of severe dysplasia showed cytoplasmic staining and the remaining cases showed membranous expression of E-cadherin (Table 3). In mild dysplasia, 16 (84%) cases showed more than 50% of cells positive but in severe dysplasia more than 50% of cases showed less than 10% of cells positive for E-cadherin expression (Table 4). With increasing grades of dysplasia, the intensity of E-cadherin was mild. All the OSMF cases showed membranous expression for E-cadherin, in which 23 (77%) cases showed suprabasal expression of E-cadherin with moderate intensity (Table 3). Out of 30 OSMF cases, 21 (70%) showed more than 50% of cells positive for E-cadherin (Table 4).

The grades of OSCC were divided into well and moderately differentiated. Among 30 cases of OSCC, 29 (97%) cases showed both membranous and cytoplasmic expression and suprabasal expression of E-cadherin (Table 3). One case did not take up the stain. In the case of well-differentiated OSCC, 11 (58%) cases showed intense staining, whereas, in moderately differentiated OSCC, nine (82%) cases showed mild intensity of E-cadherin (Table 2). Both well and moderately differentiated OSCC showed more than 50% of cells positive for E-cadherin (Table 4).

**Table 1:** Comparison of age, gender, site, and habits between the study groups Group I- normal mucosa (n=10); Group II- epithelial dysplasia (n=30); Group III- oral submucous fibrosis (n=30); Group IV- oral squamous cell carcinoma (n=30)

	GROUP I (N=10) %	GROUP II (N=30) %		GROUP III (N=30) %		GROUP IV (N=30) %		P VALUE
AGE								0.00*
21-40	9 (90%)	12 (40%)		20 (67%)		8 (27%)		
41-60	1 (10%)	15 (50%)		9 (30%)		12 (40%)		
61 AND ABOVE	0 (0%)	3 (10%)		1 (3%)		10 (33%)		
GENDER								
MALE	9 (90%)	22 (73%)		29 (97%)		21 (70%)		0.03*
FEMALE	1 (10%)	8 (27%)		1 (3%)		9 (30%)		
SITE								
BUCCAL MUCOSA	0 (0%)	25 (83%)		30 (100%)		12 (40%)		0.00*
RETROMOLAR REGION	10 (100%)	0 (0%)		0 (0%)		7 (23%)		
LATERAL BORDER OF TONGUE	0 (0%)	4 (14%)		0 (0%)		9 (30%)		
LABIAL MUCOSA	0 (0%)	1 (3%)		0 (0%)		2 (7%)		
HABITS								
NO HABITS	10 (100%)		0 (0%)		0 (0%)		4 (13%)	0.00*
CHEWING+ ALCOHOL	0 (0%)		1 (3%)		0 (0%)		1 (3%)	
CHEWING+ SMOKING	0 (0%)		3 (10%)		4 (13%)		3 (10%)	
CHEWING ALONE	0 (0%)		2 (7%)		22 (74%)		2 (7%)	
SMOKING ALONE	0 (0%)		7 (23%)		0 (0%)		6 (20%)	
ALCOHOL ALONE	0 (0%)		0 (0%)		0 (0%)		3 (10%)	
CHEWING+ SMOKING+ALCOHOL	0 (0%)		17 (57%)		4 (13%)		11 (37%)	

**Table 2:** Comparison of tissue localization, E-cadherin intensity, and basal layer intensity between the study groups Group I- normal mucosa (n=10); Group II- epithelial dysplasia (n=30); Group III- oral submucous fibrosis (n=30); Group IV- oral squamous cell carcinoma (n=30)

	GROUP I (N=10) %	GROUP II (N=30) %	GROUP III (N=30) %	GROUP IV (N=30) %	P VALUE
TISSUE LOCALISATION OF E-CADHERIN (POSITIVE STAINED CELLS)					0.00*
BASAL	0 (0%)	2 (7%)	1 (3%)	1(3%)	
SUPRA BASAL	0 (0%)	17 (57%)	23 (77%)	28 (94%)	
BASAL+ SUPRA BASAL	10 (100%)	11 (36%)	6(20%)	0 (0%)	
NEGATIVE	0 (0%)	0 (0%)	0 (0%)	1 (3%)	
E-CADHERIN INTENSITY BETWEEN THE STUDY GROUPS					
NEGATIVE	0 (0%)	0 (0%)	0 (0%)	1 (3%)	0.01*
MILD	0 (0%)	7 (23%)	10 (33%)	12 (40%)	
MODERATE	4 (40%)	16 (54%)	14 (47%)	11 (37%)	
SEVERE	6 (60%)	7 (23%)	6 (20%)	6 (20%)	
BASAL LAYER INTENSITY BETWEEN THE STUDY GROUPS					
NEGATIVE	0 (0%)	17 (57%)	23 (77%)	28 (93%)	0.001*
MILD	1 (10%)	11 (37%)	5 (17%)	2 (7%)	
MODERATE	3 (30%)	1 (3%)	2 (6%)	0 (0%)	
SEVERE	6 (60%)	1 (3%)	0 (0%)	0 (0%)	

**Table 3:** Staining pattern of E-cadherin between the study groups

Staining pattern of E-cadherin	GROUP I (N=10) %	GROUP II (N=30) %	GROUP III (N=30) %	GROUP IV (N=30) %	P VALUE
MEMBRANOUS	10 (100%)	29 (97%)	100	0 (0%)	0.00*
CYTOPLASM	0 (0%)	1 (3%)	0 (0%)	0 (0%)	
MEMBRANOUS+ CYTOPLASM	0 (0%)	0 (0%)	0 (0%)	29 (97%)	
NEGATIVE	0 (0%)	0 (0%)	0 (0%)	1 (3%)	

**Table 4:** E-cadherin-positive cells among the study groups OSCC- oral squamous cell carcinoma

	0	+1	+2	+3	P VALUE
GROUP I (N=10) %	0 (0%)	0 (0%)	0 (0%)	10 (100%)	0.00*
GROUP II (N=30) %	0 (0%)	4 (13%)	6 (20%)	20 (67%)	
GROUP III (N=30) %	0 (0%)	5(17%)	4 (13%)	21 (70%)	
GROUP IV (N=30) %	1 (3%)	5(17%)	18 (60%)	6 (20%)	
E-CADHERIN-POSITIVE CELLS IN GRADES OF DYSPLASIA					
MILD ED (n=19)%	0 (0%)	0 (0%)	3 (16%)	16 (84%)	0.02*
MODERATE ED (n=7)%	0 (0%)	1 (14%)	2 (29%)	4 (57%)	
SEVERE ED (n=4)%	0 (0%)	2 (50%)	1 (25%)	1 (25%)	
E-CADHERIN POSITIVE CELLS IN GRADES OF OSCC					
WELL-DIFFERENTIATED (n=19)%	0 (0%)	1 (5%)	12 (63%)	6 (32%)	0.03*
MODERATELY DIFFERENTIATED (n=11)%	1 (10%)	4 (36%)	6 (54%)	0 (0%)	

## Discussion

Primary epithelial cancer is characterized by excessive angiogenesis and epithelial cell proliferation during its early stages of development. The basement membrane is invaded as a result of the succeeding acquisitions of invasiveness. EMT is a crucial mechanism for the initiation of metastasis and the growth of cancer. When tight connections break down, polarity is lost, allowing the components of the apical and basolateral membranes to mix together [7].

At the adhesion junctions, E-cadherin is crucial for the growth and preservation of the epithelium [8]. Because E-cadherin's intracellular section has binding sites to interact with catenins and other regulatory proteins, its extracellular regions extend from the cell surface to bind to the presence of cadherin on adjacent cells [9]. It has been shown that the cadherin-catenin complex is disrupted in ovarian, gastric, lobular, and OSCC cancers. A marker for the prediction of metastasis, E-cadherin methylation and catenin degradation may be new targets for OSCC invasion and metastasis suppression. It has been correlated with tumor differentiation, invasion, metastasis, and prognosis [10]. One of the biggest challenges faced by healthcare professionals treating oral diseases is predicting which potentially malignant mucosal diseases (PMD), such as oral erythroplakias, leukoplakias, and lichenoid, will progress to neoplasia, specifically OSCC [11].

In OSCCS, four (13%) of patients did not have any habits. The development of malignancy in patients without habits was 40%, approximately three-fold higher than for patients with habits [12]. In the group of epithelial dysplasia, two (7%) patients did not have any habits. Both these patients were female and histopathologically classified under mild dysplasia. The white patch in these patients could be idiopathic leukoplakia, which is more prone to malignancy. On the lateral border of the tongue, OSCC is more common (Table 1) [12]. In this study, the gender distribution among the study groups showed a male predominance. These data suggest that the prevalence of OSCC is more common in males compared to females. With the prevalence of habits with respect to epithelial dysplasia, oral submucous fibrosis, and OSCC in this study, it is consistent with the finding that the combined habits of chewing, smoking, and alcohol are one of the risk factors for OPMD. The risk of oral submucous fibrosis at each betel quid chewing exposure level was stronger than that of oral leukoplakia [13,14].

**Figure 1:**
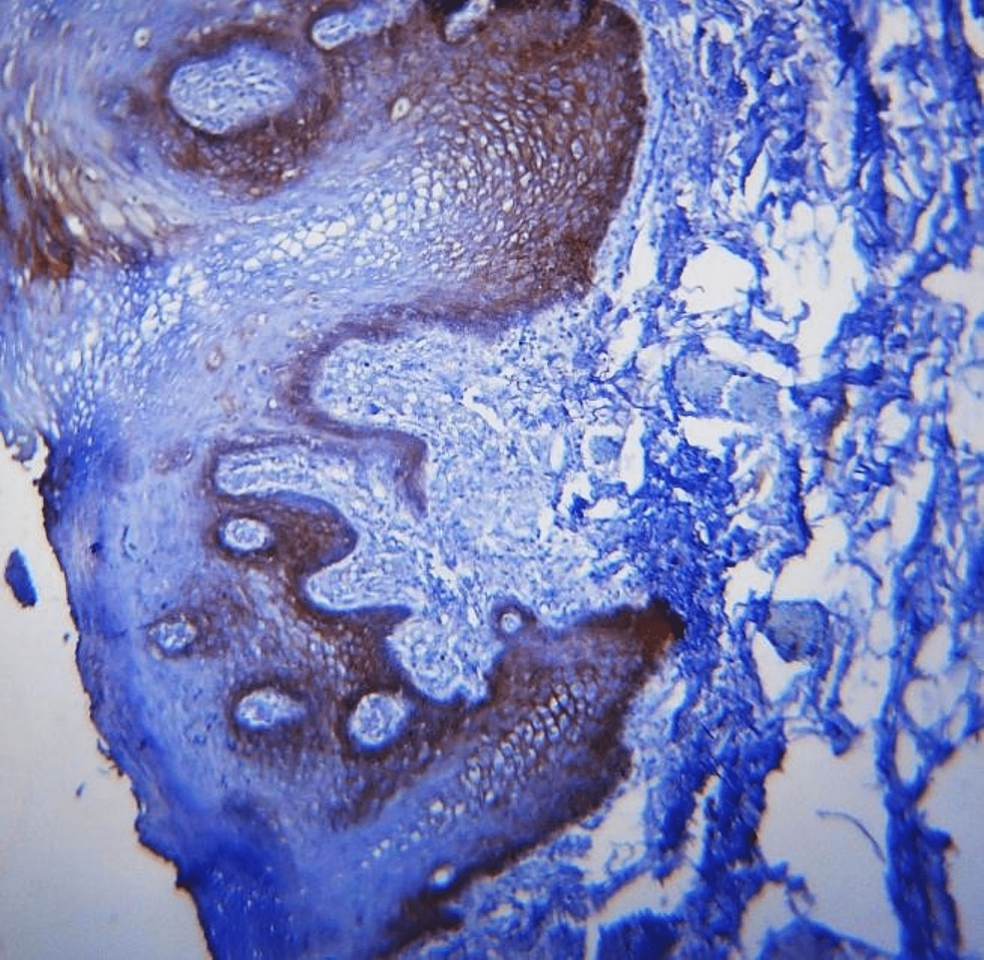
10X membranous expression of E-cadherin in leukoplakia

**Figure 2:**
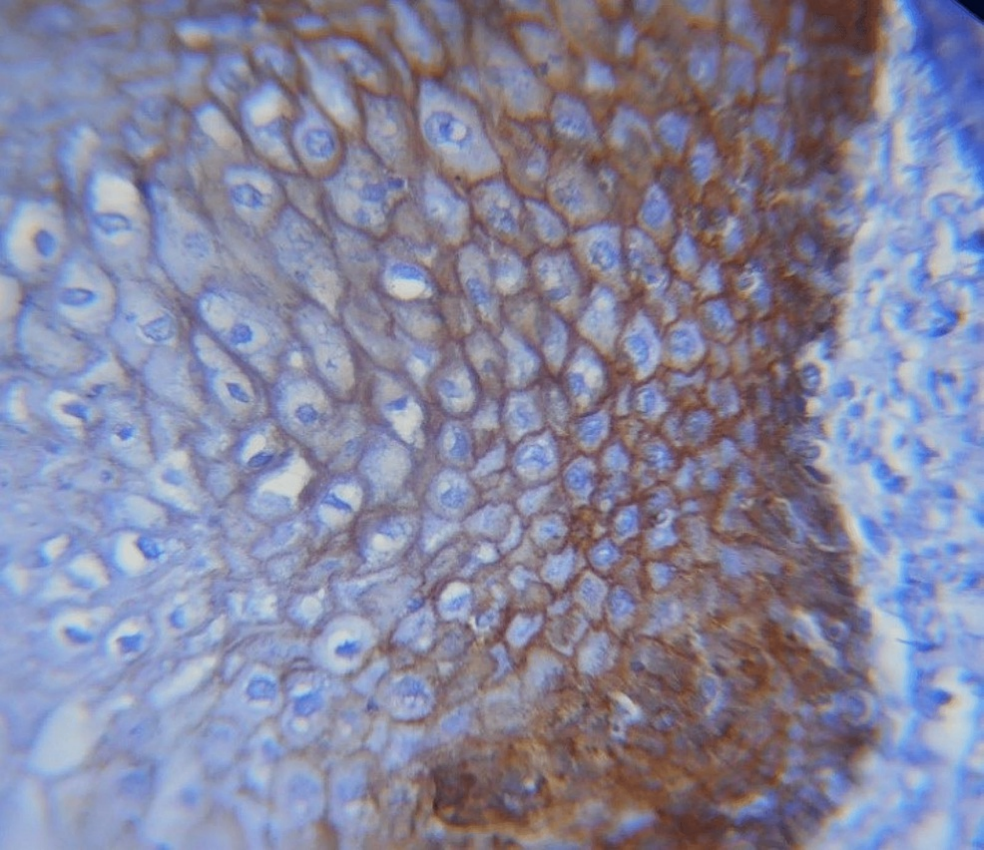
40X membranous expression of E-cadherin in leukoplakia

In this study, the E-cadherin expression in mild epithelial dysplasia was present in the suprabasal and basal layers, similar to that of normal epithelium. In moderate epithelial dysplasia, E-cadherin expression was present in the supra-basal layer but the expression was reduced in the basal cell layer (Figures 1, 2). If the basal layers of epithelium experience a loss of E-cadherin expression, it can negatively impact cellular differentiation and polarity, potentially causing cells to develop a migratory phenotype [15]. In this study, only 3% of OSCC expressed E-cadherin in the basal layer of the epithelial island, and in 3% of the cases, E-cadherin expression was not seen (Table 2). The increased rate of production and lack of mobility or adhesion to cell membranes of the protein E-cadherin may be the cause of its enhanced cytoplasmic expression in epithelial cells [16].

E-cadherin is expressed on the surfaces of normal epithelial cells. Loss of E-cadherin has been found in cancers and was postulated to facilitate tumor cell dissociation and metastasis [17]. In this study, all cases expressed E-cadherin except one case of OSCC. Sixty percent (60%) of normal mucosa cases that expressed E-cadherin showed intense staining. We further analyzed the intensity of E-cadherin stain in the three study groups and found that more than 50% of cases in the dysplasia and OSMF groups showed moderate intensity. OSMF intensity was not influenced by the duration of habits and clinical parameters such as burning sensation and mouth opening. The E-cadherin intensity was mild to moderate in dysplasia, OSMF, and OSCC compared to the severe intensity, which was seen in normal mucosa. This difference in the intensity of E-cadherin expression between the three groups compared to normal mucosa was significant (Table 2). E-cadherin expression in normal mucosa was seen in the suprabasal layer but it was absent in the keratin layer. This pattern of distribution of E-cadherin could be due to the process of normal desquamation of epithelium. The decrease in E-cadherin staining may be caused by a mutation in the E-cadherin (CDH1) gene, which could be due to CpG methylation silencing or changes in gene expression [18]. In this study, all the cases of normal mucosa expressed E-cadherin in the basal and suprabasal layers.

**Figure 3:**
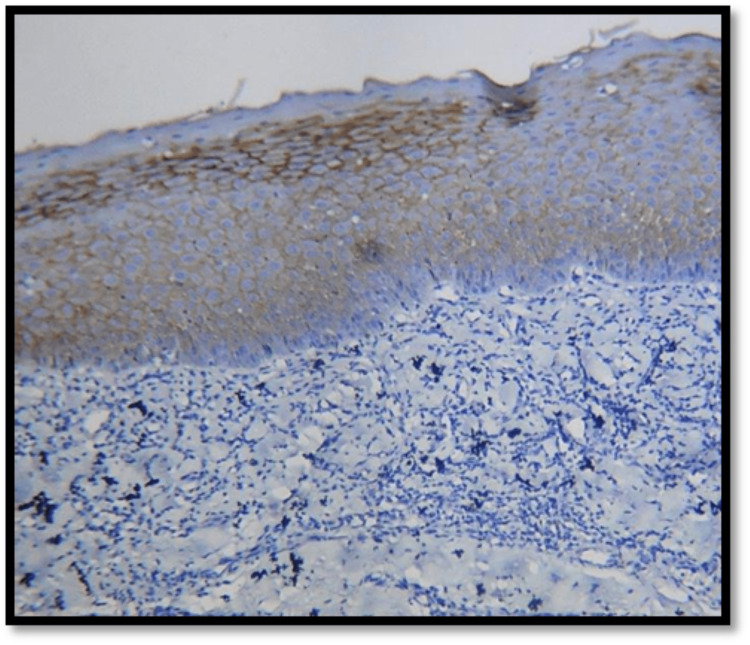
10X membranous expression of E-cadherin in OSMF OSMF- oral submucous fibrosis

**Figure 4:**
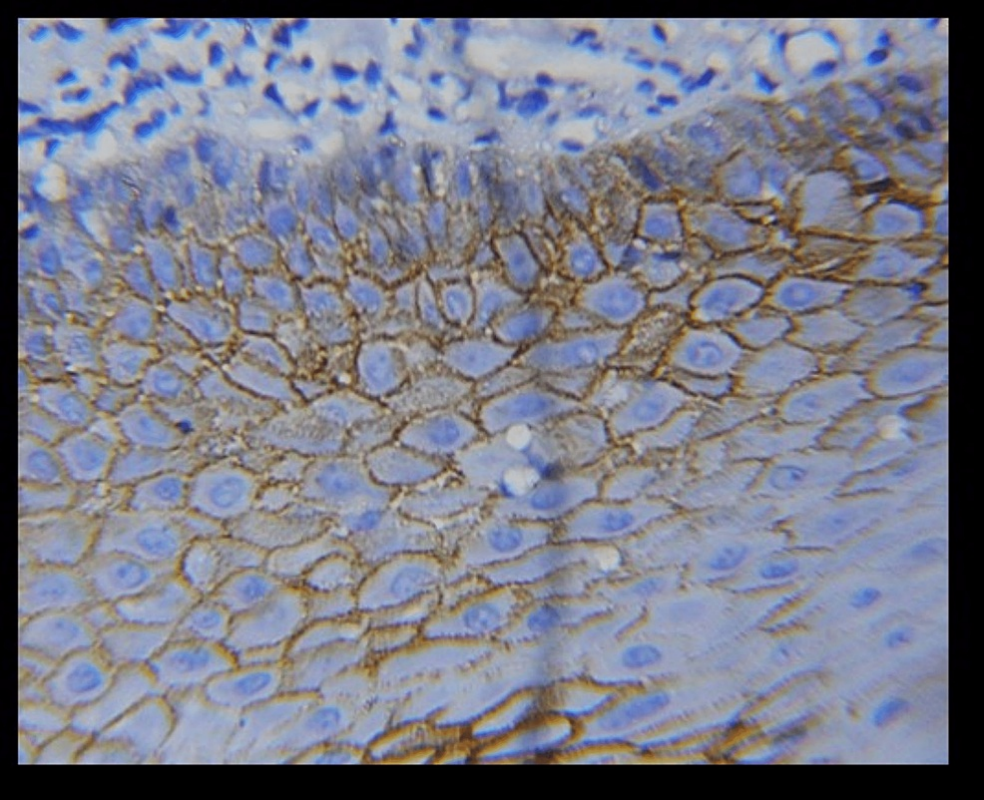
40X membranous expression of E-cadherin in OSMF OSMF- oral submucous fibrosis

**Figure 5:**
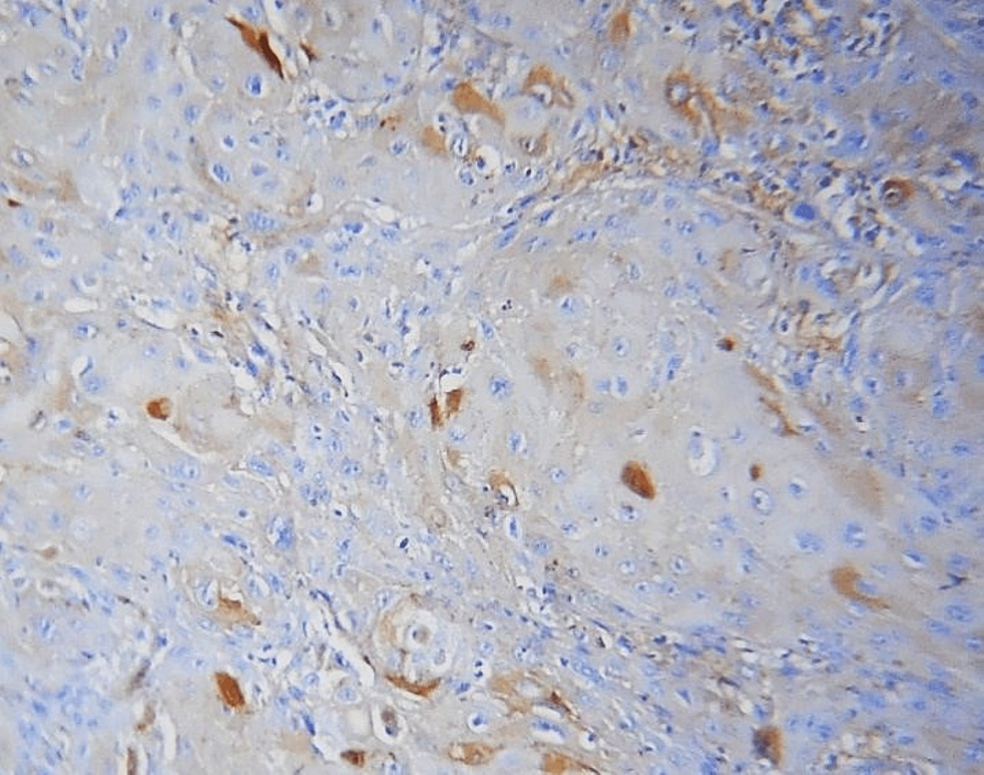
10X cytoplasmic expression of E-cadherin in OSCC OSCC- oral squamous cell carcinoma

**Figure 6:**
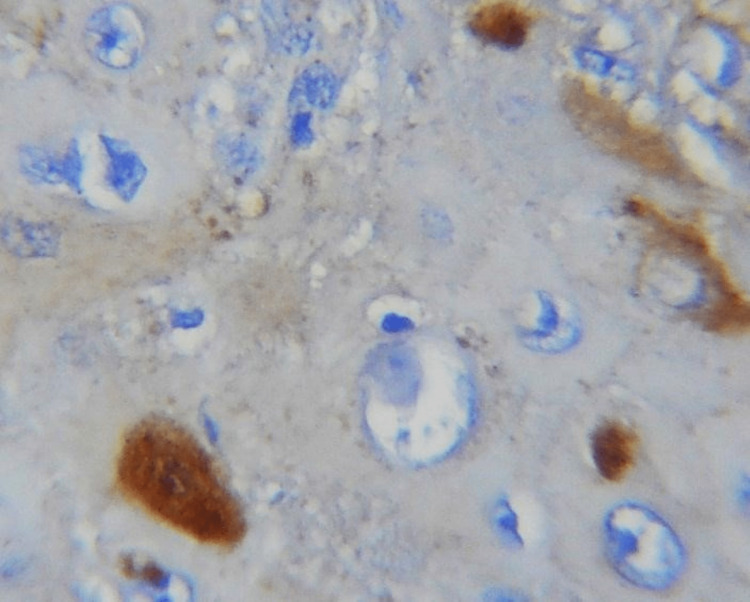
40X cytoplasmic expression of E-cadherin in OSCC OSCC- oral squamous cell carcinoma

Different E-cadherin staining patterns in dysplastic epithelium with varied degrees of dysplasia suggest that these changes may have occurred later, transitioning to an invasion-capable cell phenotype [16]. E-cadherin is localized on the surfaces of epithelial cells in regions of cell-cell contact. The normal pattern of E-cadherin expression was membranous [19]. The loss of membranous E-cadherin expression has a negative impact on cellular adhesiveness, cellular differentiation, and cellular polarity in the epithelium that induces the cells to attain a migratory phenotype, a significant feature associated with transformation [20]. In this study, all the cases of normal mucosa, dysplasia (Figures 1, 2), and OSMF showed membranous staining patterns (Figures 3, 4), and 97% of OSCC showed both membranous and cytoplasmic expression (Figures 5, 6) (Table 3) [19,20]. The enhanced manufacturing rate and lack of mobility or binding to cell membranes of the protein E-cadherin may explain its higher cytoplasmic expression in epithelial cells [21]. The pattern of E-cadherin staining was peri-cellular in distribution throughout the basal, suprabasal, and keratin layers of normal epithelium. The lower level of E-cadherin immunoreactivity in the basal and supra-basal layers could suggest issues with intracellular association, which may signal the start of the pro-carcinogenic process in the epithelial layer [15]. In this study, 60% of cases of normal mucosa expressed E-cadherin in the basal layer and the staining was intense. More than 65% did not express E-cadherin in the basal layers of dysplasia, OSMF, and OSCC. This indicates that there was a significant difference in the loss of E-cadherin in the basal layer of dysplasia, OSMF, and OSCC compared to normal mucosa. This finding highlights that the difference in the intensity of E-cadherin in OSMF and dysplasia could be used as an early marker of malignant transformation of oral potentially malignant disorders. In OSCC, E-cadherin intensity could be used as an early marker of invasiveness and metastasis [15]. This variation in the intensity could be attributed to aberrant activation of the WNT signaling component, which initiates EMT in OSCCs, leading to tumor invasiveness and metastasis [19].

On further analyzing the intensity of E-cadherin stain in the dysplasia group, there was a decrease in the intensity of E-cadherin with the increasing grades of dysplasia. Seventy-five percent (75%) of severe dysplasia cases and 53% of mild dysplasia cases expressed mild and moderate E-cadherin intensity, respectively. In this study, 58% of well-differentiated OSCC cases expressed moderate intensity of E-cadherin but 82% of moderately differentiated OSCC cases expressed mild intensity (Table 2) (Figures 5, 6). As the grade of dysplasia and OSCC increases, the intensity of E-cadherin decreases. This indicates an issue with the connections between epithelial cells, which promotes EMT during the development of OSCC [21]. Normal mucosa, dysplasia, and OSMF showed more than 50% positivity for E-cadherin expression. In OSCC, 60% of cases showed 10-50% positivity from the screened 10 high-power fields. The percentage of E-cadherin-stained cells decreased from mild to severe dysplasia (Table 4). None of the cases showed more than 50% positivity in moderately differentiated OSCC indicating the fact that E-cadherin down-regulation pathogenicity was seen in OSCC [19].

The E-cadherin expression was not seen in one case of OSCC. The reasons for the absence of staining could be attributable to SLUG-induced repression of E-cadherin, silencing of E-cadherin expression by genetic and epigenetic mechanisms, hypermethylation of E-cadherin promoter region, and transcriptional repression [22]. The proteolytic degradation of E-cadherin was mediated by MMPs, especially MMP2, MMP9, and MMP14 [23]. In cases of OSCC, the lack of E-cadherin expression may suggest that the tumor is prone to metastasizing [24]. The COX-2-dependent upregulation of Snail in inflammatory conditions reduces E-cadherin and contributes to EMT. Its higher cytoplasmic expression in epithelial cells may be due to the protein E-cadherin's increased production rate and lack of mobility or adherence to cell membranes [24]. At the front of cancer cells, the E-cadherin-mediated cell-cell adhesion system is deactivated due to tyrosine phosphorylation of Beta-catenin [25]. The establishment and maintenance of adherent junctions in regions of epithelial cell-to-cell contact depend heavily on the calcium-dependent interactions among E-cadherin molecules [26].

Observational findings in this study showed that the loss of E-cadherin in the basal layer of OSMF and dysplasia could be used as an early marker of malignant transformation. A decrease in E-cadherin intensity toward increasing grades of dysplasia and OSCC showed the reduced expression of E-cadherin, which could be used as a prognostic marker in oral potentially malignant disorders and OSCC.

The limitations of this study include that E-cadherin is not specific in diagnosing oral cancer. It can be altered in various conditions and diseases, which leads to false-positive or false-negative results so it is less reliable as a single diagnostic marker in diagnosing oral cancer. E-cadherin can be combined with p63 in the diagnosis of oral cancer.

## Conclusions

In this study, the role of E-cadherin was addressed in the EMT process in epithelial dysplasia, OSMF, and OSCC and compared with that of normal mucosa. The results of this study highlight that E-cadherin expression is reduced in increasing grades of OSCC and in increasing grades of epithelial dysplasia compared to that of normal mucosa. We also found that the staining pattern was both membranous and cytoplasmic in OSCC while there was only membranous staining in all the cases of epithelial dysplasia, OSMF, and normal mucosa. Hence, we conclude that E-cadherin can be considered a surrogate marker of malignancy.
